# Pollen season variations among anemophilous species in an Atlantic-influenced mediterranean environment: a long term study (1993–2022)

**DOI:** 10.1007/s00484-024-02796-1

**Published:** 2024-11-08

**Authors:** Nuria Montiel, Pablo J. Hidalgo, José Antonio Adame, Francisco González-Minero

**Affiliations:** 1 Department of Integrated Sciences, Centre for Natural Resources, Health and Environment (RENSMA), University of Huelva, Huelva, 21007 Spain; 2 Atmospheric Research and Instrumentation Branch, Atmospheric Sounding Station– El Arenosillo, National Institute for Aerospace Technology (INTA), Mazagón, Huelva, 21130 Spain; 3 Department of Plant Biology and Ecology, Faculty of Pharmacy, University of Sevilla, Sevilla, 41012 Spain

**Keywords:** Bioclimatology, Climate change, Airborne pollen, Huelva, Long-term trend

## Abstract

Numerous studies have shown that climate change is impacting the airborne pollen concentration of various anemophilous plant species, both herbaceous and woody. The aim of this study was to determine whether this trend, highly marked in Europe, is also occurring in the city of Huelva (south-western Spain), an area with a milder Mediterranean climate. The factors influencing the main pollen season were studied for all local taxa accounting for more than 1% of the total pollen collected, over the period 1993–2022, using a volumetric Hirst type trap to determine concentration. The study included a trend analysis of meteorological variables susceptible to climate change, specifically maximum, minimum and average temperature, precipitation and the number of hours of insolation per day. Although some of the variables analysed do not show significant trends for most taxa, such as start date or peak date, the overall results indicate a trend in the airborne pollen dynamics for herbaceous and woody taxa. Six of the eight woody taxa studied showed a significant trend towards increased pollen concentration, some of which are associated with temperature in previous seasons. In contrast, three of the five herbaceous taxa, which are more sensitive to short term meteorological variables, show a significant acceleration in the end date of the main pollen season, correlated negatively with temperature and insolation. In conclusion, some incipient changes are occurring, which may be indicative of future consequences on biodiversity in the Mediterranean area influenced by the Atlantic Ocean.

**Supplementary Information:**

The online version contains supplementary material available at 10.1007/s00484-024-02796-1.

## Introduction

Climate change is, without doubt, one of the major challenges facing the 21st century. Human-induced climate warming has led to an increase in the global average temperature of one degree Celsius above pre-industrial levels (IPCC 2021), with drastic impacts on environmental processes. Among these impacts, can be included alterations in the phenology of numerous plant species, given that phenology has been shown to be especially sensitive to temperature and rainfall (Keenan et al. 2014; Wang et al. 2015, 2022, 2023; Galán et al. 2016; Qiu et al. 2020). Under the effects of climate change, both of these variables show trends towards increasingly extreme events (IPCC 2021), and it can thus be expected that alterations in plant phenology will become more marked and more widespread. At the same time, there is evidence that an increase in atmospheric CO_2_ concentrations stimulates plant production, and consequently pollen production (Rogers et al. 2006; Albertine et al. 2014).

The interaction of all these processes in the context of climate change is generating alterations in the airborne pollen registered from numerous plant species. Variations in pollen abundance and seasonally of anemophilous species are especially identifiable, as this type of pollen has been monitored for several decades in numerous cities around the world (Ziello et al. 2012; Ziska et al. 2019). Long-term pollen monitoring in the air has enabled many studies to link alterations in airborne pollen concentration to climate change (Smith et al. 2014; Zhang et al. 2015; Anderegg et al. 2021; Kurganskiy et al. 2021). The trend in the Northern Hemisphere seems to indicate an increase in the amount of pollen recorded from woody species, such as olive (*Olea europaea*), alder (*Alnus* spp.) and hazel (*Corylus avellana*) (García-Mozo et al. 2014; Hoebeke et al. 2018) while the amount of pollen registered from herbaceous species, for instance grasses and nettles, shows a decreasing trend (Hoebeke et al. 2018; Rojo et al. 2021). In addition, significant changes in the duration of the main pollen season have been reported, with variations in the start, peak and end dates (García-Mozo et al. 2014; Hoebeke et al. 2018; Ruiz-Valenzuela and Aguilera 2018; Piotrowska-Weryszko et al. 2021; Rojo et al. 2021; Adams-Groom et al. 2022). However, it should be noted that these temporal variations seem to respond to local climatic conditions, with different results being obtained according to the region or country where the study was located. This situation makes it difficult to establish an exact and unequivocal global trend (Recio et al. 2009; Galán et al. 2016; Adams-Groom et al. 2022).

This is especially relevant for two fundamental reasons. In this context, it should be stressed that variations in pollen concentration have a direct impact on diseases such as allergic rhinitis and asthma. An increase in the amount of pollen registered and in the duration of the flowering period, in combination with an increase in atmospheric pollution, will affect both sensitisation and symptomatology (Ziska et al. 2019; Pacheco et al. 2021). Second, variations in the airborne pollen from anemophilous species is considered an indicator of the impact of climate change on ecosystem integrity and species distribution (Galán et al. 2016; Bruffaerts et al. 2018; Piotrowska-Weryszko et al. 2021; Adams-Groom et al. 2022).

This study centred on the city of Huelva, located in the southwest of the Iberian Peninsula. The area has an oceanic Termo-Mediterranean bioclimate with a strong Atlantic influence (Rivas-Martínez 1987; Valle 2004; Muñoz-Rodríguez et al. 2021). As a result, the atmospheric effects of climate change (especially temperature) can be expected to be more attenuated than in continental regions due to the maritime air masses that moderate temperatures and are less extreme. In addition, the biological composition of the air is characterized by a prevalence of species typical of both coastal and continental areas (Muñoz-Rodríguez et al. 2021). Many aerobiological studies have been carried out in the Mediterranean region, focused on long-term trends and their relationship with climate or land use changes (Damialis et al. 2007; Bonofiglio et al. 2013; Fernández-Llamazares et al. 2014; García-Mozo et al. 2014; Galán et al. 2016; Recio et al. 2018; Ruiz-Valenzuela and Aguilera 2018; Cristofolini et al. 2020; López-Orozco et al. 2021), but there are few studies in Mediterranean latitudes which cover the type of climatology directly influenced by the Atlantic Ocean (Fernández-Illescas et al. 2010; García-Mozo et al. 2010; Alcázar et al. 2011; Camacho et al. 2020). For this reason, the analysis of pollen variations in the atmosphere of Huelva is important, since the results could be representative of both the Gulf of Cádiz and the southern and western Portuguese coasts. In fact, these areas share the same biogeographical province: the Coastal Lusitan-Andalusian Province (Rivas-Martínez et al. 2017). It is characterized by common siliceous flora and vegetation, in particular degraded heather scrublands and native forests of cork and holm oaks (Rivas-Martínez 1988), while also hosting many Mediterranean Ibero-Atlantic endemism.

The main goals of this study were: (i) to explore changes in the parameters defining the main pollen season through analysis of the taxa detected in the atmosphere of Huelva; (ii) to determine whether there is a relationship between these trends and the meteorological variables involved in climate change; and (iii) to provide pollen information of use to allergenic patients and to understand the influence of pollen variations in agriculture and forestry management.

## Materials and methods

### Study area

**Fig. 1:**
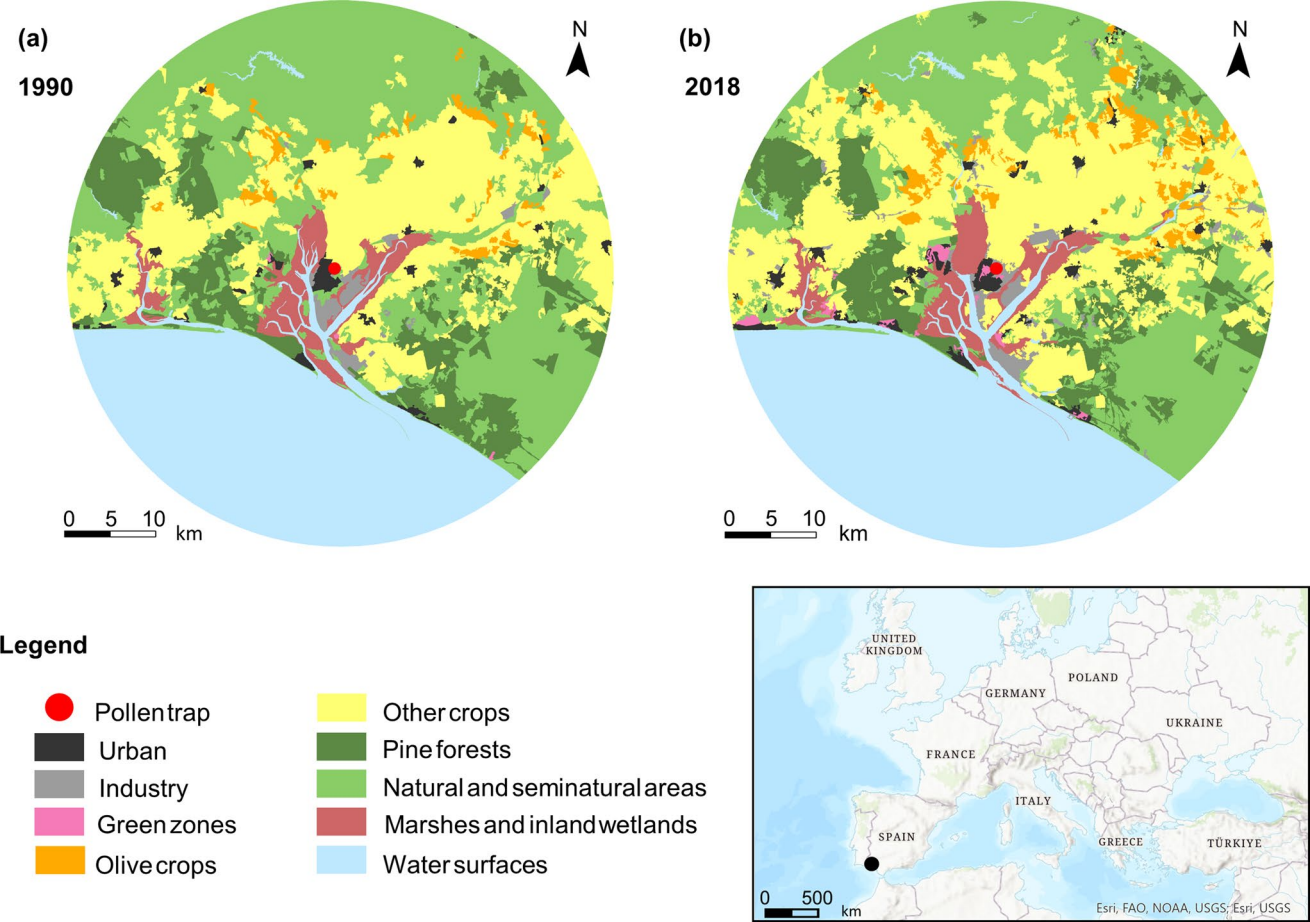
Changes in land use over a radius of 30 km around the city of Huelva: 1990 (**a**) and 2018 (**b**). Adapted from CORINE Land Cover 2020

The city of Huelva is located in the southwest (SW) of the Iberian Peninsula (37°16’N 6°57’ W), 7 km from the Atlantic Ocean as the crow flies (Fig. 1).

The area has an oceanic Mediterranean climate with average annual temperatures between 17 and 19 °C. The rainy season stretches from October to April, with an average annual rainfall of around 500–700 mm (average from 1952 to 2019) (Andalusia Regional Government 2022; State Meteorological Agency, 2023). The city is situated between the estuaries of the rivers Tinto and Odiel, such that the urban area is surrounded by an extensive marshland system. To the SW it borders the Odiel Marshes Natural Area, included in the Natura 2000 Network and considered both a Biosphere Reserve and a Ramsar Wetland, while to the east it is bordered by the Tinto Marshes and Riverside, which are also included in the Natura 2000 Network (REDIAM 2019). Consequently, a large number of species from the Amaranthaceae family can be found, such as *Arthrocnemum* sp., *Salicornia* sp. and *Sarcocornia* sp., in addition to grasses such as *Spartina* sp. that dominate these saline systems (Muñoz-Rodríguez et al. 2021). Given Huelva’s proximity to the Doñana Natural Park, the composition of airborne pollen is greatly influenced by dune systems, as well as by *dehesas* (an agrosilvopastoral system of open wooded landscapes consisting mainly of *Quercus* species), and pine forests (*Pinus pinea*). Moreover, there are large forest plantations (mainly *Eucalyptus* sp.), olive groves (*Olea europaea*) and non-irrigated herbaceous crops (Hidalgo 2021; Muñoz-Rodríguez et al. 2021). Likewise, the city of Huelva has a wide range of green spaces with native vegetation, such as holm oaks (*Quercus ilex*), cork oaks (*Quercus suber*) and junipers (*Juniperis oxycedrus*,* J. phoenicia*), in addition to ornamental vegetation and many areas that are ideal for all types of herbaceous plants (Fig. 1). In order to understand the influence of the surrounding landscape on pollen concentration, an analysis of land use changes (Katelaris et al. 2004), based on CORINE Land Cover (2020), was carried out over a 30 km radius. Changes to the surrounding areas of the city have recently been observed, in particular a relative reduction in pine forests due to wildfires (15% in 28 years) and an increase in olive crops (53% over 28 years) (Fig. 1).

### Aerobiological and meteorological observations

The aerobiological data were taken using a Hirst-type volumetric trap (Hirst 1952), which collected pollen from a rooftop 12 m a.g.l. (above ground level) at the University of Huelva’s El Carmen campus. Both the collection and the sampling procedure were carried out following the protocols of the Spanish Aerobiology Association (Galán Soldevilla et al. 2007), and fulfilled the minimum requirements established by Galán et al. (2014). The study covers the period 1993–2022.

The collected atmospheric pollen this way showed considerable biodiversity, with a total of 38 different taxa represented (Table S1, supplementary material). In order to achieve consistency across the analyses, we focused only on those taxa which accounted for more than 1% of the total pollen collected throughout the time series. This resulted in 13 taxa being selected, 8 of which corresponded to woody species and 5 to herbaceous. Before establishing the data treatment, the pollen calendars for each type were prepared, based on daily averages of the whole studied period (Fig. S2, supplementary material).

The 90% method proposed by Nilsson and Persson (1981) was used to determine the Main Pollen Season (MPS), by which the start and end of the MPS are established when the cumulative sum of pollen collected for that year reaches 5% and 95% of the total, respectively. In addition, due to the wide phenological variability of the taxa involved, it was decided that the “Peak” method should be used. This method, included in the AeRobiology R software package (Rojo et al. 2019), consists in selecting the day with the highest pollen register (the peak day) for each year and for each taxon. Once these have been established, six-month periods before and after the peak day were selected. The data from those 12 months was be used in the calculation of the MPS for each year and each taxon. The advantage offered by this technique is that it provides a more realistic analysis for certain pollen taxa, such as Cupressaceae and Urticaceae, whose pollen seasons usually cover two different years. The MPS parameters analysed in this study were: start day, end day, peak day, value for peak day, length of the MPS, SPIn (Seasonal Pollen Integral), calculated as the sum of the daily averages during the MPS, and APIn (Annual Pollen Integral) (Fig. S3, supplementary material), calculated as the sum of the daily averages for the whole 12-month period (Galán et al. 2017; Bastl et al. 2018). For missing data, the default method recommended in the AeRobiology R package was followed: where these data did not exceed 30 consecutive days, linear interpolation was used (always ensuring that this did not influence the MPS); in cases where this threshold was exceeded, the gap in the data series was maintained (Rojo et al. 2019). The resulting dataset covered the period from 1993 to 2022, with some years omitted from the study (Table 1, column “N”) whenever the availability of data was insufficient to encompass the full pollen season.

To understand how weather conditions affect pollen, daily surface meteorological observations were used, specifically, the maximum (Tmax), minimum (Tmin) and average (Tmean) temperatures, rainfall (Rain), and daily insolation (Insol). The meteorological records were obtained from the state meteorological observatory in Huelva (State Meteorological Agency (AEMET), 2023) which is located 1.5 km from the pollen monitoring station. This database covers the period from 1992 to 2022, for which daily averages for each season and variable were taken. Winter was defined as January to March, spring from April to June, summer from July to September and autumn from October to December.

### Statistical analysis

First, the mean and standard deviation were calculated for all aerobiological parameters, after which the Shapiro-Wilk normality test was applied. This indicated that the majority of the results could not be considered normal, and so non-parametric tests were used for the subsequent analyses. The calculation of the trends for both the aerobiological and meteorological parameters was conducted using the Mann-Kendall test and the Theil-Sen estimator (positive Theil-Sen values indicate an increasing trend), as these statistical tools are considered more robust for heterogeneous time series data. Special attention was paid to the p-value (p), indicating the significance of the analysis, and the Theil-Sen estimator, which states the value of the slope of the regression line (Hussain and Mahmud 2019).

The relationship between the aerobiological variables and the meteorological measurements was analysed using a Spearman correlation test, which resulted in two statistical parameters: the p-value, indicating the significance of the test, and the Rho value, indicating the type of correlation (Rho > 0: positive correlation; Rho < 0: negative correlation) and its strength (Rho = 0: no correlation; Rho = 1: perfect correlation (Virtanen et al. 2020). The correlations were made using the aerobiological variables for each taxon, and the seasonal average for the meteorological variables for the entire duration of the MPS, plus two seasons prior to the season in question to understand the possible influence on the parameters of the MPS.

As mentioned above, the MPS parameters were generated using the AeRobiology R package (Rojo et al. 2019). All statistical analyses and graphics were performed using Python (v.3.11). The packages and libraries used were pyMannKendall (Hussain and Mahmud 2019) for the Mann-Kendall test, SciPy (Virtanen et al. 2020) for the Spearman correlation test and the Theil-Sen estimator, and Matplotlib (Hunter 2007) and Seaborn (Waskom 2021) for the graphs.

## Results

### Aerobiological pollen overview

Table 1 shows the main results for the aerobiological parameters over the time series. Regarding the duration of the main pollen season, there is considerable variation. For some taxa, notably Amaranthaceae and Myrtaceae, the MPS last several months, probably due to the diversity of species represented, whilst others, such as the plane tree (*Platanus*) and olive tree (*Olea*), represented by a single species, it is considerably shorter, at approximately one month.

**Table 1:** Taxa accounting for more than 1% of total pollen collected from the air of Huelva

Taxa		*N*	Start date		Peak date		End date		Lenght		APIn		SPIn		Peak value	
Woody	*Casuarina*	24	266	± 12	282	± 19	332	± 23	68	± 26	291	± 175	268	± 161	51	± 43
	Cupressaceae	21	310	± 30	48	± 37	99	± 32	156	± 50	1587	± 1224	1444	± 1113	108	± 86
	Myrtaceae	22	101	± 41	181	± 11	267	± 38	167	± 62	461	± 349	418	± 316	31	± 27
	*Olea*	23	114	± 9	125	± 13	158	± 16	44	± 11	4352	± 3135	3976	± 2875	532	± 426
	*Pinus*	23	58	± 20	105	± 18	180	± 45	123	± 55	741	± 506	674	± 458	81	± 76
	*Platanus*	23	67	± 10	76	± 7	96	± 16	30	± 16	977	± 911	907	± 856	161	± 130
	*Populus*	21	35	± 39	65	± 21	94	± 24	60	± 50	268	± 496	249	± 456	40	± 87
	*Quercus*	23	60	± 44	97	± 11	141	± 16	82	± 43	4593	± 3374	4190	± 3101	492	± 380
Herbaceous	Amaranthaceae	22	110	± 10	219	± 47	276	± 6	167	± 12	1549	± 664	1403	± 601	71	± 60
	*Plantago*	23	84	± 10	111	± 18	174	± 25	91	± 28	611	± 433	556	± 393	44	± 41
	Poaceae	23	102	± 23	139	± 9	204	± 27	104	± 43	4282	± 3860	3870	± 3494	330	± 354
	*Rumex*	23	53	± 16	101	± 27	153	± 20	101	± 28	671	± 500	609	± 453	35	± 24
	Urticaceae	22	362	± 23	71	± 18	151	± 38	155	± 41	2703	± 2407	2441	± 2176	118	± 121

Average and standard deviation for all aerobiological parameters over the time series. Start, peak and end dates, and length of MPS are expressed in DOY (days of the year). APIn (Annual Pollen Integral), SPIn (Seasonal Pollen Integral) and peak value are expressed in pollen*day/m^3^. N indicates the numbers of years used in the analyses for each taxon (see Sect. 2.2)

With respect to the amount of pollen, there are also large variations between the different taxa. In terms of SPIn, the taxa contributing the largest amounts are *Quercus*, *Olea*, Poaceae and Urticaceae, while those contributing the least are *Casuarina* and *Populus*.

Finally, it is worth noting the standard deviation of certain taxa with respect to the start and end dates of the MPS. For example, in the case of Myrtaceae, *Quercus*, *Populus* or *Pinus*, one, or even both, shows a variation of up to one and a half months, while for other taxa the deviation is negligible, as can be seen in the case of Amaranthaceae, the start and end dates of which are always around the same week of the year.

### Trends in the aerobiological parameters

Table 2 shows the Theil-Sen slope values and slope deviation for the pollen season parameters. With respect to start dates of the MPS, neither the woody or herbaceous species seem to show significant trends, with the exception of the Poaceae group (*p* = 0.034), which shows a trend towards a delay in the start date. With regard to peak day, the most significant trend is shown by *Casuarina* (*p* = 0.015), which tends towards a delay in the peak date. Regarding the end date, however, the herbaceous taxa show considerable alteration. As can be seen in Table 2, the end date is significantly advanced for three out of the five taxa, resulting in a reduction in the MPS. This is especially evident in the Poaceae group, whose MPS length is the most significantly reduced (*p* = 0.001).

The differences between herbaceous and woody taxa are once again notable in terms of the amount of pollen collected (APIn, SPIn, and peak day value) (Table 2). In this case, *Casuarina*, *Olea*, *Pinus*, *Platanus* and *Populus* show a significant increase in pollen concentration, while Myrtaceae, shows a decreasing trend. Herbaceous taxa, on the other hand, do not seem to show any changes in their pollen concentration, with the exception of Urticaceae, which shows a reduction for SPIn (*p* = 0.017).

**Table 2:** Theil-Sen slope values and slope deviation for the pollen season parameters of woody and herbaceous taxa measured in Huelva (1993–2022)

	Woody taxa								Herbaceous taxa				
	*Casu.*	Cupr.	Myrt.	*Olea*	*Pinu.*	*Plat.*	*Popu.*	*Quer.*	Amar.	*Plan.*	Poac.	*Rume.*	Urti.
Start date											*		
	0.9	13.4	-18.6	-1.6	1.7	0.4	-4.7	3.3	0.0	2.1	7.1*	2.5	-3.1
	± 6.9	± 16.0	± 24.4	± 4.7	± 6.6	± 3.9	± 16.2	± 12.6	± 5.8	± 6.2	± 9.4	± 10.1	± 13.5
Peak date	*												
	11.8	0.6	0.0	2.4	5.6	0.0	-4.3	5.0	4.0	5.0	-3.3	8.0	-2.5
	± 8.6	± 14.1	± 7.2	± 7.8	± 8.5	± 3.9	± 10.0	± 5.0	± 8.2	± 9.8	± 5.6	± 15.4	± 12.5
End date										+	**	*	
	2.7	9.7	17.6	-4.2	-0.6	-0.8	0.0	4.4	2.0	-7.6	-20.0	-5.0	-15.0
	± 7.9	± 9.6	± 25.6	± 8.1	± 29.1	± 7.7	± 13.1	± 9.7	± 3.3	± 8.9	± 10.0	± 5.8	± 23.3
Length		+								*	**		
	3.2	-13.3	27.3	-1.9	-1.7	-1.0	10.0	0.9	2.5	-10.0	-31.4	-9.2	-12.0
	± 12.7	± 31.5	± 39.6	± 5.4	± 26.3	± 5.3	± 20.6	± 16.3	± 6.5	± 11.5	± 14.4	± 13.5	± 23.5
APIn	+		***	**	*	**	***						*
	80.4	378.5	-174.0	1808.5	288.4	547.3	113.6	578.3	22.8	-3.3	-384.0	-77.7	-914.6
	± 87.2	± 510.5	± 103.4	± 1247.3	± 229.3	± 336.5	± 115.4	± 1431.8	± 425.4	± 183.6	± 1628.7	± 245.4	± 806.3
SPIn	+		***	**	*	**	***						*
	76.4	344.0	-154.0	1668.5	271.1	482.5	105.8	531.3	22.9	-7.037	-344.5	-70.0	-824.1
	± 79.0	± 487.2	± 95.3	± 1116.4	± 208.1	± 314.8	± 108.1	± 1285.5	± 384.3	± 167.0	± 1482.7	± 212.0	± 731.6
Peak value			**	**	*	*	***						
	7.0	35.4	-11.4	243.1	32.1	60.0	10.0	46.3	9.4	1.9	0.455	-6.0	-12.0
	± 13.5	± 35.0	± 9.9	± 157.0	± 32.9	± 50.2	± 9.2	± 166.7	± 18.3	± 8.1	± 112.3	± 8.5	± 33.4

Start date, peak date, end date and length of MPS are expressed in days decade^-1^. APIn (Annual Pollen Integral), SPIn (Seasonal Pollen Integral) and peak values are expressed in pollen decade^-1^. The p-value is expressed as follows: + *p* = 0.05; * *p* < 0.05; ** *p* < 0.01; *** *p* < 0.001. The taxa are: Casuarina (Casu.), Cupressaceae (Cupr.), Myrtaceae (Myrt.), Olea, Pinu (Pinus), Platanus (Plat.), Populus (Popu.), Quercus (Quer.), Amaranthaceae (Amar.), Plantago (Plan.), Poaceae (Poac.), Rumex (Rume.) and Urticaceae (Urti.)

### Trends in the meteorological parameters

**Fig. 2:**
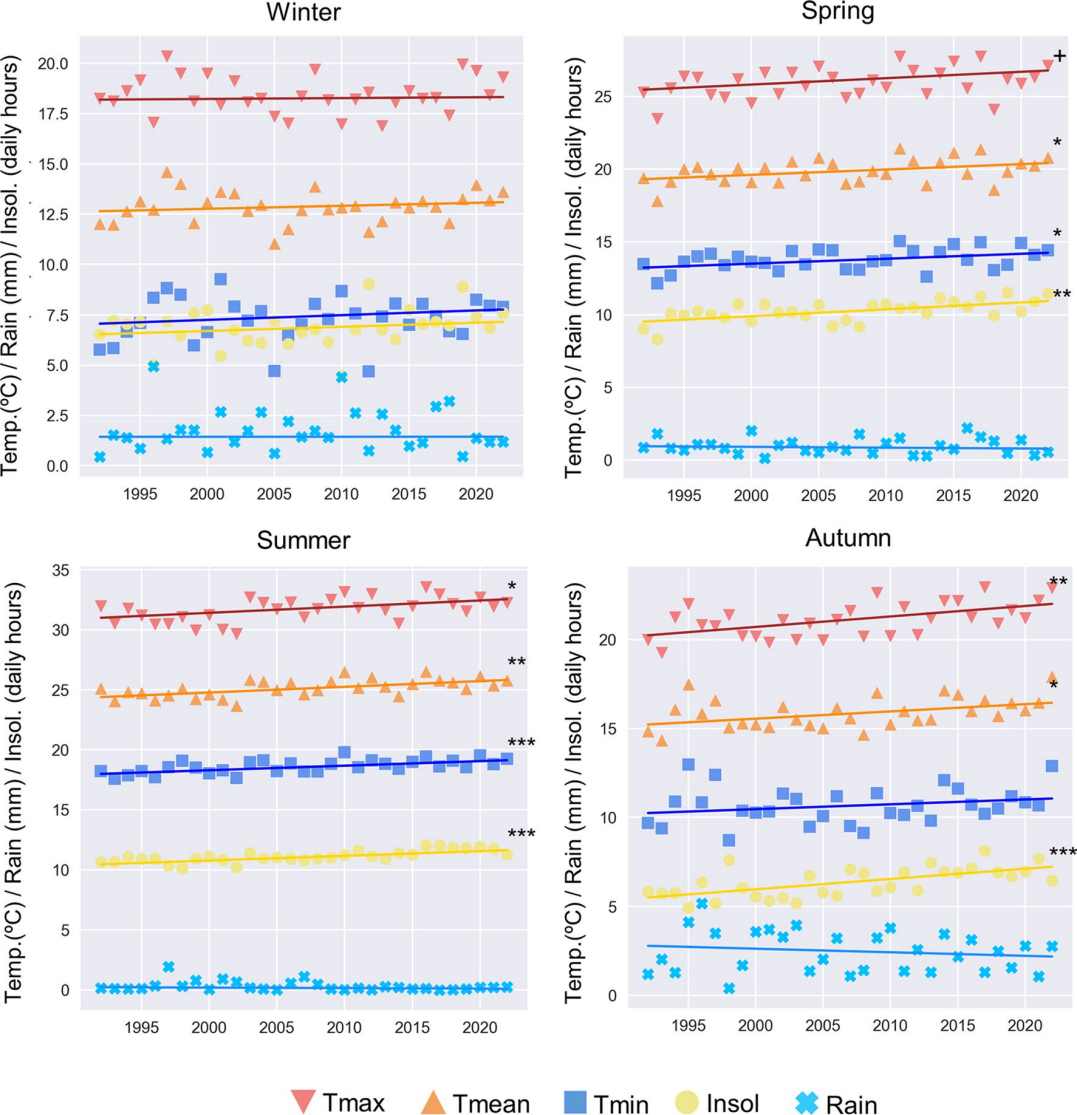
Temporal evolution of the seasonal mean values of the meteorological parameters and their trend line (1992–2023). The p-value is expressed as follows: + *p* = 0.05; * *p* < 0.05; ** *p* < 0.01; *** *p* < 0.001. The seasons are divided into winter (JFM), spring (AMJ), summer (JAS) and autumn (OND). The meteorological variables are daily averages for each season: Tmax (maximum temperature), Tmean (average temperature), Tmin (minimum temperature), Insol. (Insolation) and Rain (rainfall)

Table 3; Fig. 2 show the main variables likely to be affected by climate change. With the exception of rainfall, all show significant changes over time, especially in spring (SMJ), summer (JAS) and autumn (OND). In the case of rainfall, a negative trend can be detected over the years, especially in the case of autumn, albeit not significant.

**Table 3:** Theil-Sen slope values and slope deviation for meteorological variables by season in Huelva (1992–2022)

Seasons	Tmax.		Tmean.		Tmin.		Rain		Insol.		
Winter	0.12	± 1.4	0.6	± 1.3	0.9	± 1.8	0.0	± 1.6	0.8	± 1.6	
Spring	1.8	± 1.8	1.5	± 1.4	1.4	± 1.2	-0.2	± 0.9	1.9	± 1.0	
Summer	2.1	± 1.6	1.9	± 1.0	1.5	± 0.8	-0.2	± 0.4	1.6	± 0.7	
Autumn	2.4	± 1.4	1.6	± 1.2	1.1	± 1.7	-0.8	± 1.9	2.3	± 1.1	

Maximum (Tmax), minimum (Tmin) and average (Tmean) temperatures are expressed in ºC season decade^-1^, rainfall (Rain) is expressed in mm season decade^-1^ and insolation (Insol) is expressed in h/day season decade^-1^

### Correlation between aerobiological and meteorological parameters

Given that the main variations were found in the length of the MPS, end date, and pollen concentration (SPIn, APIn and peak value), analysis of potential correlations with the meteorological variables focused on these three parameters. As the trends for APIn and SPIn were similar, SPIn was selected over APIn for its advantage in temporally delimiting the analysis.

**Fig. 3:**
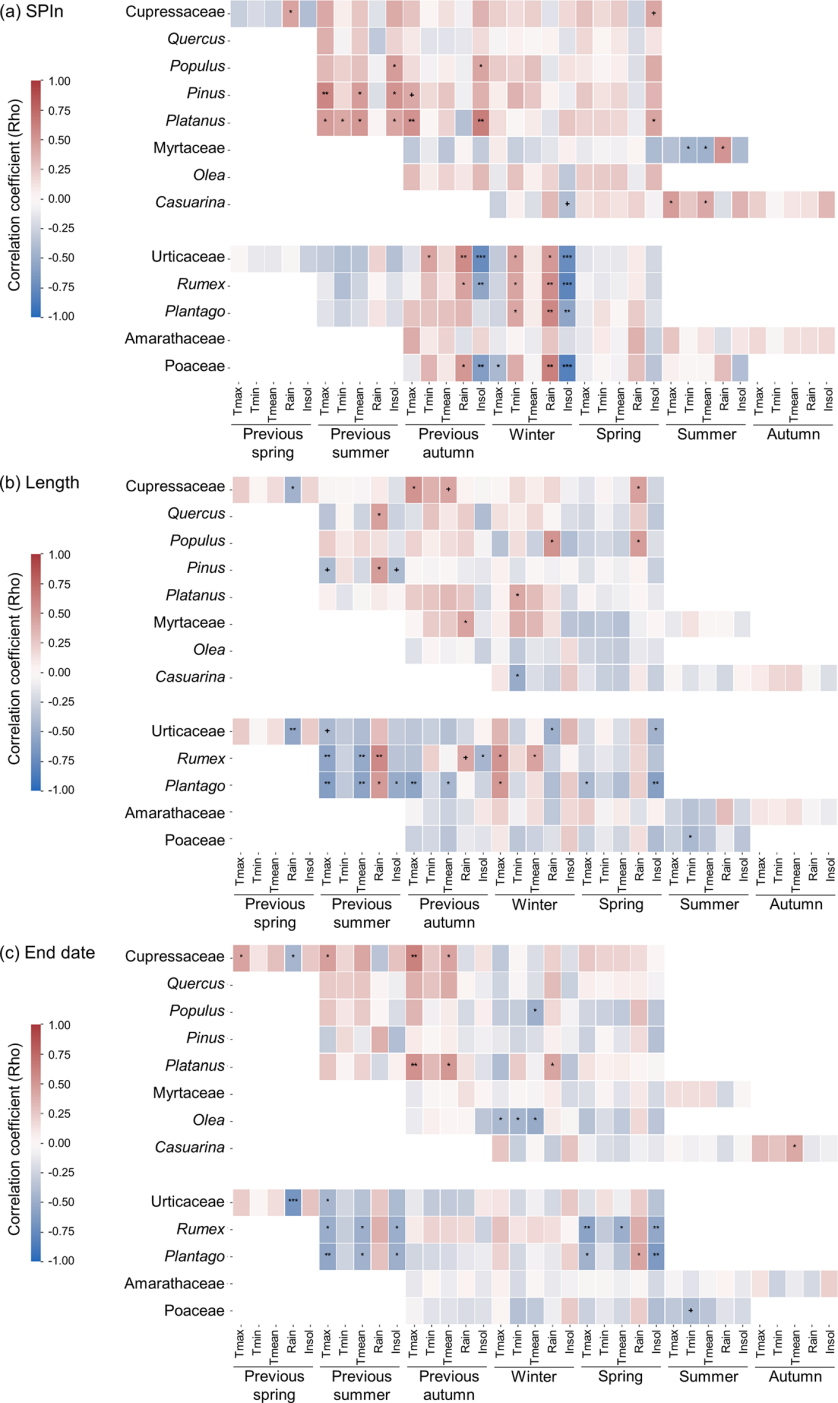
Results of the Spearman correlation of (**a**) SPIn, (**b**) length, (**c**) end date with seasonal meteorological variables for woody and herbaceous taxa. The p-value is expressed as follows: + *p* = 0.05; * *p* < 0.05; ** *p* < 0.01; *** *p* < 0.001. The meteorological variables are Tmax (maximum temperature), Tmean (average temperature), Tmin (minimum temperature) expressed in ºC, Insol. (Insolation) expressed in daily hours, and Rain (Rainfall) expressed in mm

With regard to woody taxa, some show a direct correlation between SPIn and temperature and insolation of the season prior to the flowering period (Fig. 3a). The case of Myrtaceae is worth noting, as its pattern is inverse to the rest of the woody taxa, showing a negative correlation between SPIn and summer temperature. By contrast, a highly significant correlation can be detected among the herbaceous taxa between hours of insolation and rainfall and the pollen concentration. A further positive correlation can be seen between this pollen concentration and winter minimum temperatures for Urticaceae, *Rumex* and *Plantago*.

The correlation between the length of the MPS and the meteorological variables (Fig. 3b) once again shows that there is a large difference between the results obtained for herbaceous and woody plants. In the case of woody taxa, rainfall is the factor which correlates most strongly with length of MPS, followed by temperature. Herbaceous taxa, on the other hand, show a significant correlation with maximum and average temperatures, and with rainfall, depending on the taxa.

With respect to the correlation between the end of the pollen season and the meteorological variables (Fig. 3c), the results for *Rumex* and *Plantago* are particularly noteworthy. The end date closely correlates with temperature and insolation (and rainfall in the case of *Plantago*) at the end of the pollen season. With regard to arboreal taxa, it seems that there is no marked correlation with meteorological variables, except in the case of Cupressaceae, *Olea* and *Platanus*, where the end date correlates with the temperature of the previous month.

## Discussion

In this study, a comprehensive analysis of the main airborne pollen in Huelva was carried out using time series data from 1993 to 2022. The city has some particularities in that it is surrounded by a large marshland system and is located in an area of oceanic Mediterranean climate with a strong influence from the Atlantic Ocean. Data analysis identified significant trends in both pollen concentration (especially with respect to woody taxa) and in the various parameters that define the MPS (particularly in the case of herbaceous taxa). In addition, several of these trends correlated with meteorological variables that are undergoing alterations due to climate change.

The huge phenological variability of the taxa in the Huelva air, alongside a comparable variability in their composition, should also be highlighted. Many of these taxa, such as Poaceae, are composed of many species, each with a different phenology (Devesa et al. 2020). This heterogeneity added an extra layer of complexity to the study and obliged us to reflect more deeply on the results obtained.

First, over the study period there is a significant increase in the concentration of airborne pollen detected for *Platanus*, *Populus*, *Olea*,* Casuarina* and *Pinus*. The positive trends in SPIn found for four of these five taxa have already been reported in other regions, both in the Mediterranean area and in higher latitudes: *Olea* (Damialis et al. 2007; Galán et al. 2016; Ruiz-Valenzuela and Aguilera 2018), *Platanus* (Damialis et al. 2007; Alcázar et al. 2011; Galán et al. 2016; Ruiz-Valenzuela and Aguilera 2018), *Populus* (Makra et al. 2011) and *Pinus* (Damialis et al. 2007; Fernández-Llamazares et al. 2014; Galán et al. 2016; De Linares et al. 2017; Ruiz-Valenzuela and Aguilera 2018). However, these taxa-specific trends do not seem to follow a general pattern, and respond strongly to local situations, differences even being found within the same area by different studies (Galán et al. 2016; De Linares et al. 2017; Ruiz-Valenzuela and Aguilera 2018). Such differences could be due in part to land use changes in the proximity of the pollen sampler or to situations governed by local microclimates.

The SPIn of these woody taxa depends chiefly on temperature and daily hours of insolation in the months leading up to the start date. This effect, which has already been described both for temperature (Ziska et al. 2019; López-Orozco et al. 2021; Adams-Groom et al. 2022) and for daily hours of insolation in the case of *Quercus* (Recio et al. 2018; López-Orozco et al. 2021), *Betula* (Hoebeke et al. 2018; De Weger et al. 2021) and *Platanus* (Hoebeke et al. 2018), is also documented in the present study. This finding shows that, despite being a location close to the ocean, where the buffering effect of the body of water plays an essential role, the effects of climate change on pollen concentration are significant, especially for certain taxa such as *Pinus* and *Platanus* (both of which display a positive trend for SPIn, and a correlation of this parameter with the temperature of previous seasons).

In the case of *Olea*, although there is an evident correlation with temperature, it is not statistically significant. The increase in SPIn for this taxon may be caused by a combination of the increase in temperature and changes in land use. Analysis of the cultivation of this type of crop in the agricultural area surrounding Huelva reveals that from 1990 to 2018 land cover increased by 53% within a radius of 30 km around the position of the pollen trap (CORINE Land Cover 2020). In addition, as other researchers have pointed out, the use of irrigation with this type of crop is associated with the disappearance of the biennial rhythm that formerly characterised the production and emission of pollen in olive trees (Díaz de la Guarda et al. 2003). This circumstance may be contributing to the positive trend found in the SPIn and APIn (Díaz de la Guarda et al. 2003; Galán et al. 2016). Although the MPS of the olive tree is very limited in time (44 days ± 11 days), *Olea* pollen is among the most allergenic, and an increase in emission can have a negative impact on the health of people sensitive to this allergen (Bonofiglio et al. 2013). In *Pinus* forests, an opposite pattern was found, whereby pollen concentration has increased despite a reduction– due mainly to wildfires– in the surface area of this type of forest within a 30 km radius of the pollen trap (in fact, the period 1990–2018 saw a reduction in the area of pine forest of 15%, Fig. 1, CORINE Land Cover 2020). Hence, the increase in the SPIn for *Pinus* pollen can be considered as a possible consequence of the increase in temperature during the months leading up to flowering.

Myrtaceae, on the other hand, display a significant negative trend with respect to SPIn. Although an a priori negative correlation with summer temperatures can be assumed, another significant factor is that the *Eucalyptus* sp. plantations in areas around the city, formerly supplying a now decommissioned paper mill, are being replaced by other types of crops (Caetano Sánchez et al. 2017).

In the case of Cupressaceae and *Quercus*, no significant trends were found for SPIn over the study period. These findings are consistent with research carried out in other areas of the Iberian Peninsula (Fernández-Rodríguez et al. 2016; Galán et al. 2016), although other studies in the vicinity have found contrasting results (Fernández-Llamazares et al. 2014; Ruiz-Valenzuela and Aguilera 2018; López-Orozco et al. 2021). Studies conducted elsewhere in the Mediterranean region, also show an increasing trend in pollen concentration for these taxa (Damialis et al. 2007). In this regard, we can conclude that half of the woody taxa analysed in this study follow a general trend in the Northern Hemisphere towards an increase in pollen concentration (García-Mozo et al. 2014; Bruffaerts et al. 2018; Ruiz-Valenzuela and Aguilera 2018; Rojo et al. 2021). Nevertheless, a closer look at this trend in terms of specific taxa shows that in some cases the general pattern is diluted in response to regional variations, whether as a result of local climate, or other factors such as variations in land use (García-Mozo et al. 2016). It is also worth mentioning that, to our knowledge, no studies have been found that consider the trends in the airborne pollen of *Casuarina* or Myrtaceae.

The herbaceous taxa show a detectable trend in the values for SPIn, although (with the exception of Urticaceae) these are not statistically significant. Nevertheless, there is no agreement on the trend for this taxon (Urticaceae), some researchers reporting an increase in pollen (Damialis et al. 2007; Makra et al. 2011), others finding an absence of any trend (Bruffaerts et al. 2018; Ruiz-Valenzuela and Aguilera 2018; Manangan et al. 2021). However, other areas of the Iberian Peninsula have reported results similar to ours (Recio et al. 2009; Ruiz-Valenzuela and Aguilera 2018). As many studies point out, herbaceous plants are much more sensitive to short-term climatic factors, having an almost immediate response (Galán et al. 2016). The results of our study indicate that the SPIn for Urticaceae (in addition to that of Poaceae, *Rumex* and *Plantago*, though not statistically significant) is closely correlated with rainfall and daily hours of insolation, and to a lesser degree with temperature. It is well known that an increase in rainfall stimulates pollen production in herbaceous taxa (Rojo et al. 2021), while a rainy and humid environment decreases pollen dispersal, so a balanced study of both is the key to understanding the whole dynamics (Hoebeke et al. 2018). With respect to solar radiation, there is evidence that in woody taxa it stimulates pollen production (Hoebeke et al. 2018; Recio et al. 2018; De Weger et al. 2021; López-Orozco et al. 2021), a finding which is corroborated for some of the woody taxa in this study, while in herbaceous taxa, it has a moderate influence (positive or negative) depending on the taxon (Majeed et al. 2018). In our case, insolation has a negative effect on herbaceous species, in terms of both the SPIn (a decrease) and the end date (earlier). As shown in Fig. 2, insolation increased significantly in Huelva over the course of the study period, which had a negative effect on herbaceous species. Thermal stress due to more radiation during the early phenophases of blooming could be the reason of this negative effect.

On the other hand, regarding the parameters defining the length of the MPS, there appears to be a lack of any clear trends among the woody taxa. Of particular note is the start date for Cupressaceae, as our findings contrast strongly with the trend towards an earlier start described for this taxon in both the Iberian Peninsula and other areas of Europe (Ruiz-Valenzuela and Aguilera 2018; Cristofolini et al. 2020; Rojo et al. 2021). This disparity could be due to the difference in methods used. While previous studies have used the data from a calendar year (January to December) for each MPS, the present study uses the peak method recently proposed by Rojo et al. (2019). This method provides a much closer fit to reality, as the analysis for calculating the pollen season can be extended beyond December or before January, rather than being restricted to a calendar year.

In contrast, among the herbaceous taxa we can see a general advance in the end date of the MPS, especially significant in the case of *Rumex*, Poaceae and *Plantago*. With respect to *Rumex*, other studies have found that the start is becoming delayed and the duration of the MPS shortened (Damialis et al. 2007), although yet others report a significant advance in the start date (Makra et al. 2011). In the case of Poaceae, our data indicates that in addition to an advance in the end date, this taxon is undergoing a delay in the start date. These findings contrast with those of other studies carried out across Europe, which have observed a general advance in the start date, while the end date is delayed or no significant trend can be determined (Makra et al. 2011; Hoebeke et al. 2018; Rojo et al. 2021). Finally, in the case of *Plantago*, other studies report an earlier start to the MPS (Makra et al. 2011) and an increase in its duration (Damialis et al. 2007). A more recent study, however, found a delay in the start, an earlier end, and a shortened duration for this taxon (Ruiz-Valenzuela and Aguilera 2018).

Due to the phenological variability of herbaceous taxa, in conjunction with their tendency to respond immediately to meteorological variables (Galán et al. 2016), finding a common pattern among them is far more complex than with the woody taxa. For this reason, when considering these plants it is much more logical to attend to local patterns. In the case of the city of Huelva, we can see that the early end to the MPS correlates with the increase in temperature and insolation in spring, a correlation which is particularly strong in the case of *Rumex* and *Plantago*. Similar correlations with temperature have also been reported for herbaceous taxa in nearby regions, including *Plantago* (Ruiz-Valenzuela and Aguilera 2018). Poaceae pollen shows the most marked trend towards a reduction in its MPS. This is particularly notable because, despite this shortening of the MPS, no variations in SPIn were detected for this taxon. The inference to be drawn is that the concentration of pollen over the shortened period is more intense and, consequently, people sensitive to this type of pollen are likely to show worse symptoms.

The reduction in length of the MPS for herbaceous taxa would seem to be partially associated to high insolation in the final months of their MPS. Besides, a rainfall and temperatures in the months before the start date (previous summer (JAS) previous autumn (OND) and winter (JFM)) seem to affect the MPS length. Table 1 shows that all herbaceous taxa start their MPS between December and April. It means that climatic conditions previous to this period are decisive for early phenophases that finally affect the start of pollination and MPS length. In the case of Amaranthaceae, this family seems to be less dependent on environmental variables (see Fig. 3), showing lower variation in the parameters that define its MPS (see Table 1).

Finally, the findings with respect to Amaranthaceae should be highlighted. As a family growing in abundance in the marshes around the urban area, it is highly characteristic of the airborne pollen in Huelva and an important taxon to monitor. The results indicate that all the values defining its MPS are essentially stable over studied period. Variation among the parameters is almost non-existent, comparable only to *Olea* and *Platanus*, both of which are represented by a single species. Other studies, whether in the Iberian Peninsula or in other areas of Europe, describe variations of Amaranthaceae in both APIn and SPIn (Damialis et al. 2007; Makra et al. 2011; Piotrowska-Weryszko et al. 2021), or in the parameters that define the MPS (Damialis et al. 2007; Makra et al. 2011; Ruiz-Valenzuela and Aguilera 2018; Piotrowska-Weryszko et al. 2021). Nevertheless, there are also studies, like ours, in which this taxon does not show significant variations (Fernández-Llamazares et al. 2014; Galán et al. 2016; Manangan et al. 2021). It should be noted, however, that the main source of pollen for this group, a community of Amaranthaceae in the Huelva salt marshes, has a completely different phenology to other related species of the same family. The Amaranthaceae community located in the salt marshes is mainly composed of species such as *Sarcoconia* sp., *Salicornia* sp., *Arthrocnemum macrostachyum*. and *Halimione* sp. These species, characteristic for low, medium and high marshes (Muñoz-Rodríguez et al. 2021), are dependent on brackish water and less dependent on other climatic variables as other terrestrial species of Amaranthaceae, such as *Amaranthus* sp. or *Alternanthera* sp. (Castroviejo 1993) and, therefore, more resilient to possible effects of climate change. A previous study linked daily pollen concentrations for this group with variations in wind speed and solar radiation over a two-year period (Fernández-Illescas et al. 2010). However, in the present study we were unable to establish any statistical relationship with the climatic parameters.

## Conclusions

From the analyses we carried out, it can be concluded that the airborne pollen in Huelva is undergoing an incipient alteration. More than half the woody taxa show a trend towards increasing SPIn values (*Casuarina*, Myrtaceae, *Olea*, *Pinus*, *Platanus* and *Populus*). In contrast, the trend for most of the herbaceous taxa is towards an early end to their MPS (*Plantago*, Poaceae and *Rumex*), although no variation was found in pollen concentrations (with the exception of Urticaceae, which tended towards a decrease). In conclusion, the responses observed in this study are notable in both woody and herbaceous taxa. These changes seem to be partly attributable to changes in the global and regional climates, but also partly to changes in land use. With respect to climate change, the results found in this study represent a first step towards understanding how these alterations in climatic variables can modify plant ecosystems in regions with this type of climate: the Mediterranean region with Atlantic influence.

## Electronic supplementary material

Below is the link to the electronic supplementary material.

Supplementary Material 1

Supplementary Material 2
