# Supplementary figures and images for: Pollen season variations among anemophilous species in an Atlantic-influenced mediterranean environment: a long term study (1993–2022)
Source: Int J Biometeorol. 2024 Nov 8;69(1):109–22. doi: 10.1007/s00484-024-02796-1 (PMC11680626; doi:10.1007/s00484-024-02796-1)

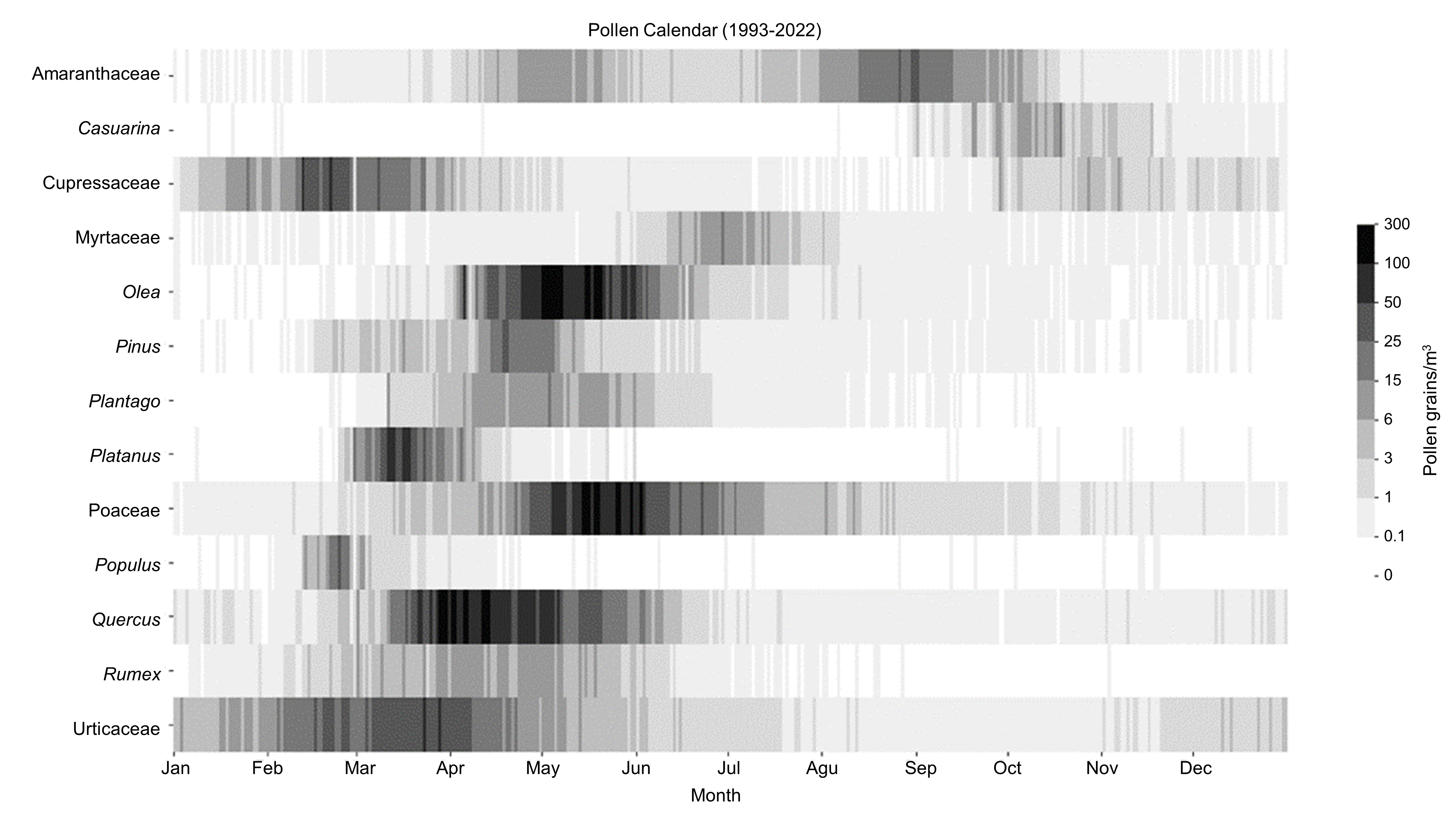

Supplement: Supplementary file 1 — Supplementary Material 1 [file 484_2024_2796_MOESM1_ESM.tif]

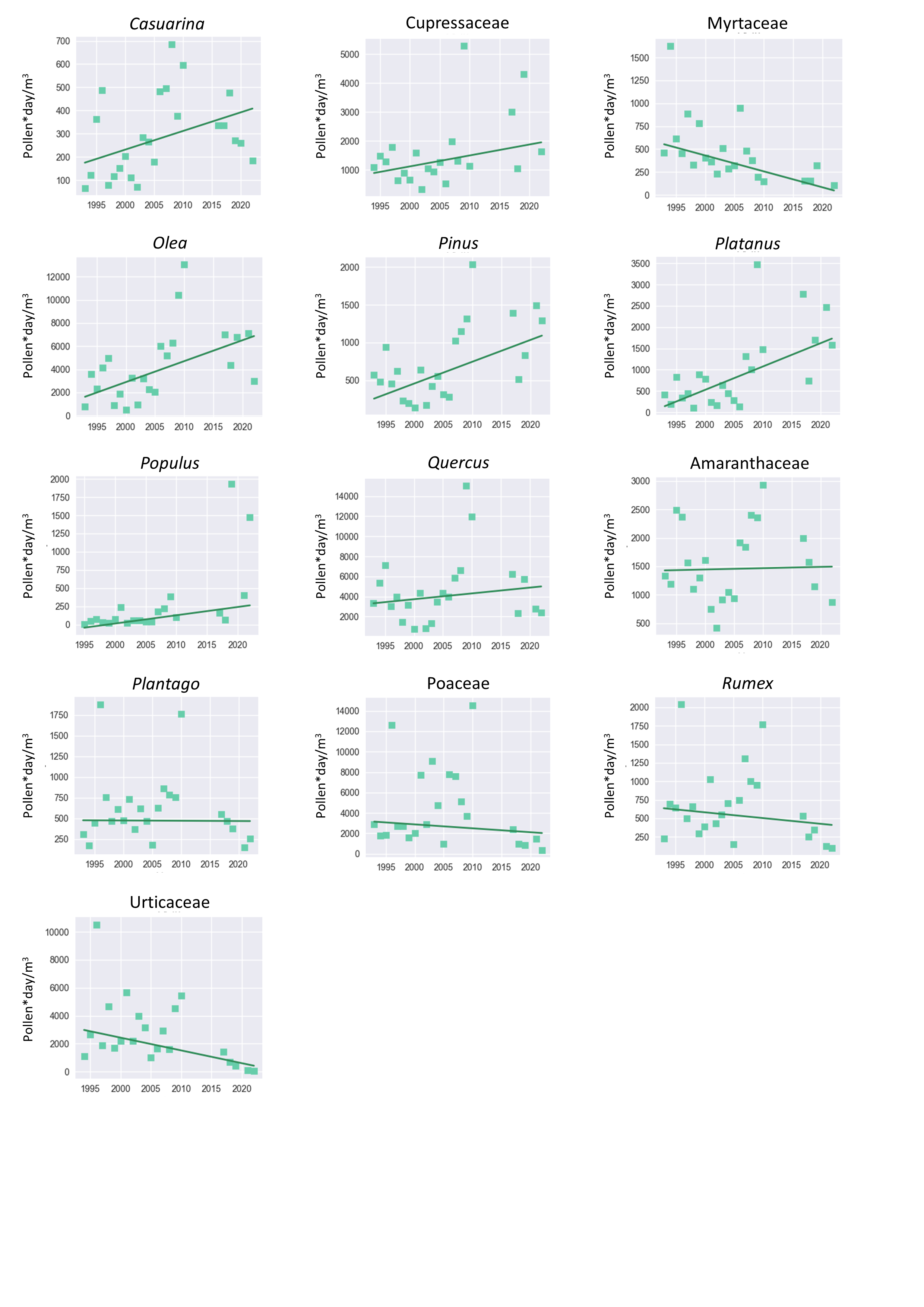

Supplement: Supplementary file 2 — Supplementary Material 2 [file 484_2024_2796_MOESM2_ESM.tif]
